# Touch influences perceived gloss

**DOI:** 10.1038/srep21866

**Published:** 2016-02-26

**Authors:** Wendy J. Adams, Iona S. Kerrigan, Erich W. Graf

**Affiliations:** 1 Psychology, University of Southampton, Southampton, SO17 1BJ, ENGLAND

## Abstract

Identifying an object’s material properties supports recognition and action planning: we grasp objects according to how heavy, hard or slippery we expect them to be. Visual cues to material qualities such as gloss have recently received attention, but how they interact with haptic (touch) information has been largely overlooked. Here, we show that touch modulates gloss perception: objects that feel slippery are perceived as glossier (more shiny).Participants explored virtual objects that varied in look and feel. A discrimination paradigm (Experiment 1) revealed that observers integrate visual gloss with haptic information. Observers could easily detect an increase in glossiness when it was paired with a decrease in friction. In contrast, increased glossiness coupled with decreased slipperiness produced a small perceptual change: the visual and haptic changes counteracted each other. Subjective ratings (Experiment 2) reflected a similar interaction – slippery objects were rated as glossier and vice versa. The sensory system treats visual gloss and haptic friction as correlated cues to surface material. Although friction is not a perfect predictor of gloss, the visual system appears to know and use a probabilistic relationship between these variables to bias perception – a sensible strategy given the ambiguity of visual clues to gloss.

Humans are proficient at distinguishing different object materials, e.g. metal, glass and plastic, based upon their visual appearance^1^, an important skill for object recognition and for guiding interaction with the environment. Whilst material perception appears effortless, the underlying computations are complex and under-constrained: an object’s image is determined not only by how its surface reflects light, but also by the object’s shape and the structure of the illumination field. In recent years, one aspect of material perception – visual gloss – has been widely studied^2^. Glossy and matte surfaces can be differentiated using specular highlights; bright image patches that occur when light is reflected from a surface regularly, in a mirror-like way, rather than scattered diffusely^3^,^4^,^5^,^6^,^7^,^8^.

Glossiness is conceptualised as a visual property. In contrast, other material dimensions have been studied within the haptic (touch) domain, such as surface roughness, thermal conductivity, compliance (or softness, i.e. deformation in response to force) and slipperiness (related to the coefficients of friction)^9^,^10^,^11^. However, little is known about how visual and haptic information interact when we estimate material properties, with existing research almost entirely constrained to the perception of roughness^12^,^13^,^14^,^15^,^16^,^17^,^18^ with a few studies on the visual-haptic cues to compliance^19^,^20^,^21^,^22^,^23^,^24^. Here we ask whether the feel of an object also affects our perception of its glossiness.

A substantial body of work has demonstrated the perceptual benefit of combining vision and haptic information when humans estimate geometric attributes such as surface slant or object size. Visual and haptic information is integrated optimally: judgments are more precise (less variable) for visual-haptic stimuli than when based on either vision or haptics alone^25^,^26^. However, this contrasts with multi-sensory findings in material perception: although some visual-haptic averaging has been found in surface roughness perception, studies have not found improvements in precision when both modalities provide information, relative to a single modality^12^,^15^. Moreover, robust contrast (repulsion) effects – the opposite of integration - have been found within material perception. For example, in the material-weight illusion, when two sized-matched objects appear to be made of different materials (e.g. balsa wood vs. metal), the denser-looking object feels lighter, when both are lifted and have the same mass^27^,^28^. Similarly, in the size-weight illusion, when two objects appear to be constructed of the same material, but differ in size, the larger object feels lighter when both are lifted and have the same mass^29^,^30^. Standard models of sensory integration predict the opposite perceptual effects^31^.

Here we describe two experiments that investigate whether humans integrate visual gloss information with haptic ‘rubberiness’ information when judging material properties. In our experiments, ‘glossier’ objects have higher specular reflectance, and less specular scatter than less glossy objects (see Table 1). We define ‘rubbery’ objects (e.g. squash balls) as those with high friction and compliance. In contrast, non-rubbery objects (i.e. a snooker ball) have low friction and high stiffness (low compliance), and can be described as feeling slippery and hard (see Table 1). Our experimental set-up allows us to present visual-haptic objects that can vary independently along these visual and haptic dimensions (Fig. 1). Visual and haptic stimuli were matched in size, shape and location, giving observers the compelling sense that they are touching and viewing the same object. Experiment 1 employed a discrimination paradigm to determine whether our participants’ ability to distinguish different visual-haptic objects is consistent with (i) independent processing of visual and haptic signals, or (ii) integration of visual and haptic cues. In Experiment 2, we used a completely different, but complementary paradigm to further explore visual-haptic interactions: observers were presented with a single visual-haptic object per trial, and gave subjective ratings of a) how glossy each object looked, as well as b) how slippery and c) how hard the object felt.

## Results

### Experiment 1

Here we report our participants’ ability to discriminate between stimuli that differ in glossiness and rubberiness. On each trial, observers viewed and touched three visual-haptic stimuli and identified which one was ‘made of something different’. The manner in which the internal estimates of these visual and haptic signals interact and influence discrimination will reflect the degree to which they have been correlated during our previous interactions with the environment^32^. For example, humans have learnt that the felt size of an object is highly correlated with its visually-estimated size, and these two signals are, therefore, fully integrated to form a single, visual-haptic size estimate^25^.

**Figure 1:**
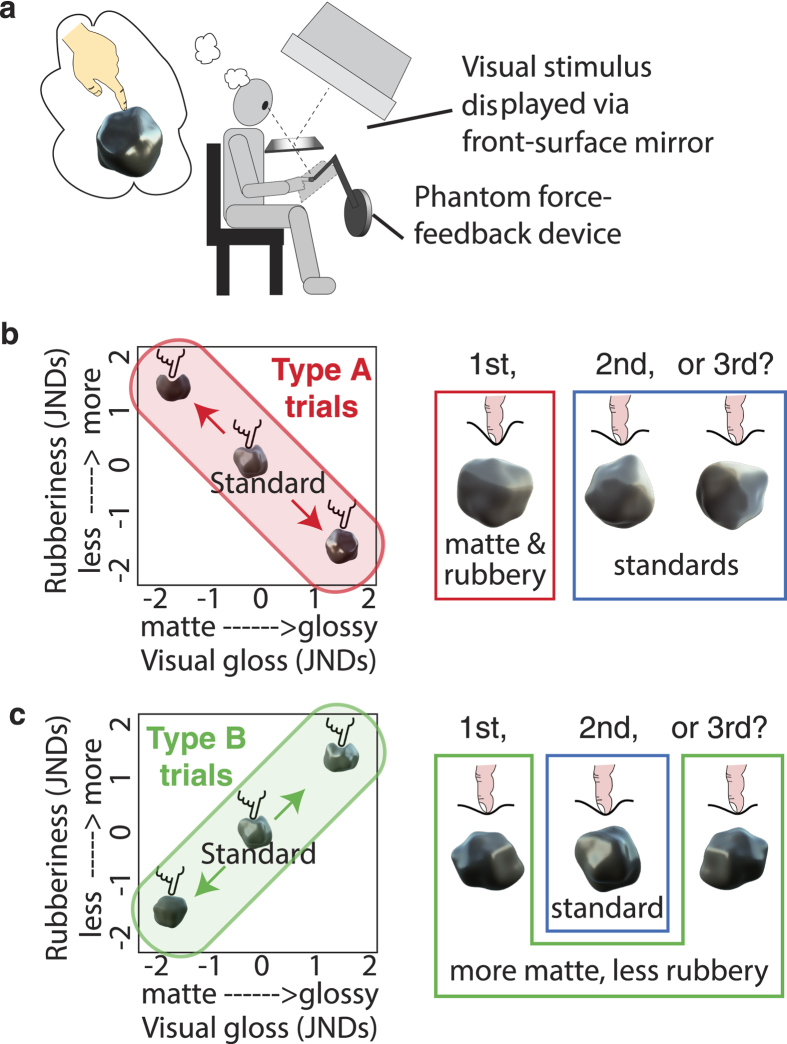
Experiment 1 stimuli. (**a**) The visual-haptic set-up and an example stimulus. (**b**) Stimuli for Type A trials lay along the negative diagonal: i.e. visual gloss and haptic rubberiness were negatively correlated. An example trial is shown to the right, see Methods for details. (**c**) The stimulus axis for Type B trials, with an example trial.

The visual and haptic cues to material properties that we consider here are unlikely to be perfectly correlated in the environment; we can conceive of matte and glossy objects that feel equally rubbery. However, we hypothesise that on average, objects that feel more rubbery will look *less* glossy, i.e. there is a negative correlation between glossiness and rubberiness for objects that we experience in our environment. In other words, objects that we encounter in our environment might not be uniformly distributed across the stimulus parameter space, but be clustered around the negative correlation axis (Fig. 1b). If observers have learnt such a correlation, this should result in partial integration^32^,^33^, which will be reflected by our observers’ ability to discriminate between visual-haptic objects that differ in gloss and rubberiness (see Methods for a detailed explanation of the discrimination predictions). In brief, stimuli that vary along the visual-haptic stimulus axis shown in Fig. 1b (Type A stimuli), in which glossiness and rubberiness are negatively correlated, will be relatively easy to discriminate. Stimuli that vary along the orthogonal axis (Type B stimuli, Fig. 1c) will be harder to discriminate. In other words, if observers have learnt a correlation between vision and haptic material cues, cross-modal integration will shift visual and haptic estimates towards the learnt visual-haptic mapping.

**Figure 2:**
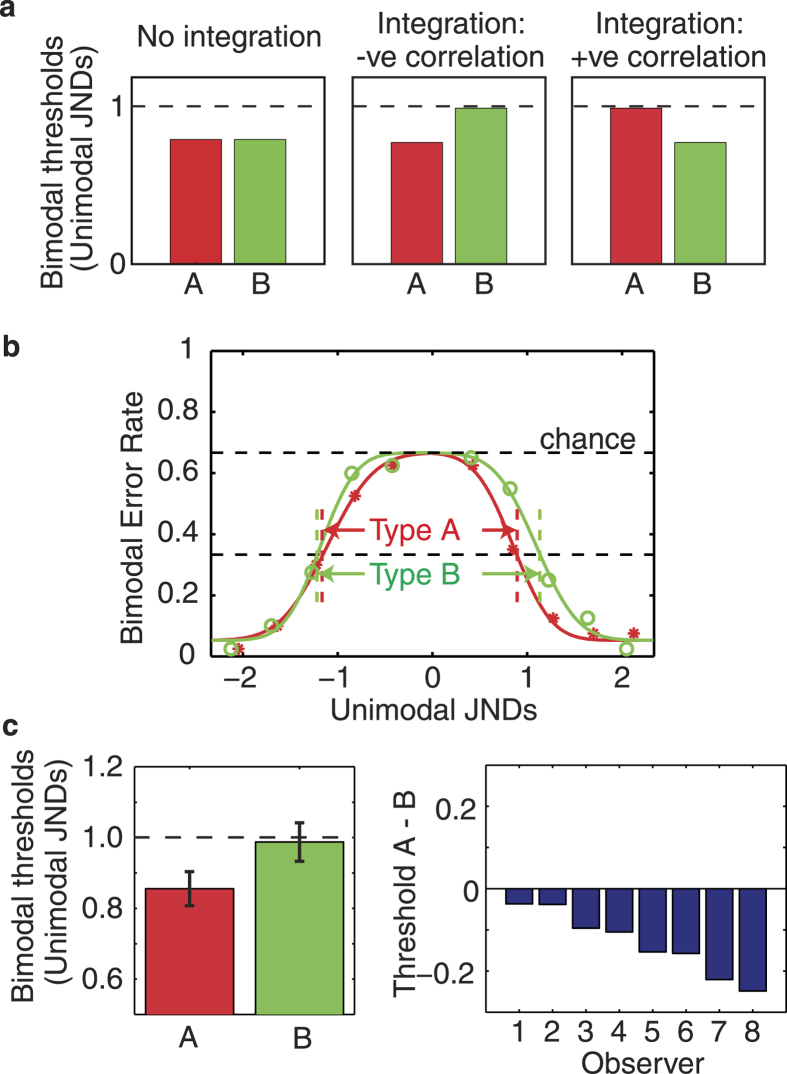
Experiment 1 Predictions and Results. (**a**) Hypothetical discrimination performance for Type A (red) and Type B (green) bi-modal stimuli (i) in the absence of integration (left), (ii) given integration driven by a negative gloss-rubberiness correlation (middle) or (iii) integration driven by a positive correlation (right). (**b**) Error rates and fits for Type A & B trials for one naïve observer. (**c**) Left: Averaged (N = 8) threshold data (mean ± 1 SE). Right: Difference between Type A and Type B thresholds for all 8 observers.

Figure 2a shows three possible experimental outcomes – predicted visual-haptic discrimination thresholds given three different integration behaviours:

(i) No integration: If the visual and haptic dimensions are unrelated in the environment, then the two signals should not be integrated for perception. Independent processing of the visual and haptic estimates will produce identical thresholds for Type A and Type B stimuli. Because the observer can identify the odd stimulus based on vision and/or haptics, she/he will have a two-cue advantage, relative to uni-modal thresholds (1 JND). In the absence of integration we predict thresholds of 0.78 JNDs for both Type A and Type B trials (see Methods: Procedure, Experiment 1 for a full explanation of discrimination predictions).

(ii) Partial integration, negative correlation: If observers have learnt that visual gloss and haptic rubberiness are negatively correlated, as described above, then we expect smaller thresholds for Type A than for Type B stimuli – the magnitude of this difference depends on the strength of integration (see Methods).

(iii) Partial integration, positive correlation: If observers have learnt that visual gloss and haptic rubberiness are positively correlated, i.e. that glossy stimuli feel more rubbery, on average, than matte stimuli, then discrimination will be better (smaller thresholds) for Type B than Type A stimuli.

Error rates for the bi-modal discrimination task for one naïve subject are shown in Fig. 2b. Each subject’s data were fit with cumulative Gaussians using a single lapse rate (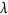, corresponding to the lower asymptote), and an upper asymptote of 0.67 (chance performance: upper dashed line in Fig. 2b). Four separate mean (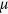) and slope (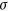) parameter pairs were used to fit the data either side of the standard, for both Type A and Type B trials. These fits were used to estimate thresholds at the 0.33 error rate (i.e. halfway between chance and perfect performance: lower dashed line in Fig. 2b). This method provided excellent fits to the data (*r*^2^ = 0.95 ± 0.007 across observers). The two thresholds for each trial type were averaged, with the resultant Type A and Type B mean thresholds shown in Fig. 2c, averaged across observers (left plot) and for individual observers (right plot). All observers show better discrimination for Type A than Type B trials, consistent with a negative learnt association between visual gloss and haptic rubberiness, i.e. matte objects more often feel rubbery, whereas glossy objects are more likely to feel hard and slippery.

### Experiment 2

Experiment 1 showed that visual gloss and haptic rubberiness are partially integrated - our sensory system has learnt that glossy objects are more likely to be slippery and hard than sticky (high in friction) and compliant, and the visual and haptic signals are integrated accordingly. However, in Experiment 1, compliance and friction always co-varied – thus we cannot determine whether the discrimination effects were driven by the integration of visual gloss with friction, or with compliance or both. Experiment 2 addressed this question and also asked whether the visual-haptic integration suggested by discrimination performance in Experiment 1 is also evident in subjective judgements of perceived gloss, compliance and friction.

In each trial, observers rated the gloss, friction and compliance of a single visual-haptic object. Each of these three stimulus attributes varied independently across trials. To determine which of these visual and haptic stimulus dimensions had a reliable effect on our subject’s ratings we used leave-one-out cross-validation to compare regression models comprised of all possible combinations of single predictors and their interactions for each rating type. To preview the key results: gloss ratings were predicted by stimulus gloss and stimulus friction. In complement to this, friction ratings were predicted by both stimulus friction and stimulus gloss. However, compliance and gloss appear to be processed independently – neither modulated perception of the other.

#### Gloss Ratings

**Figure 3:**
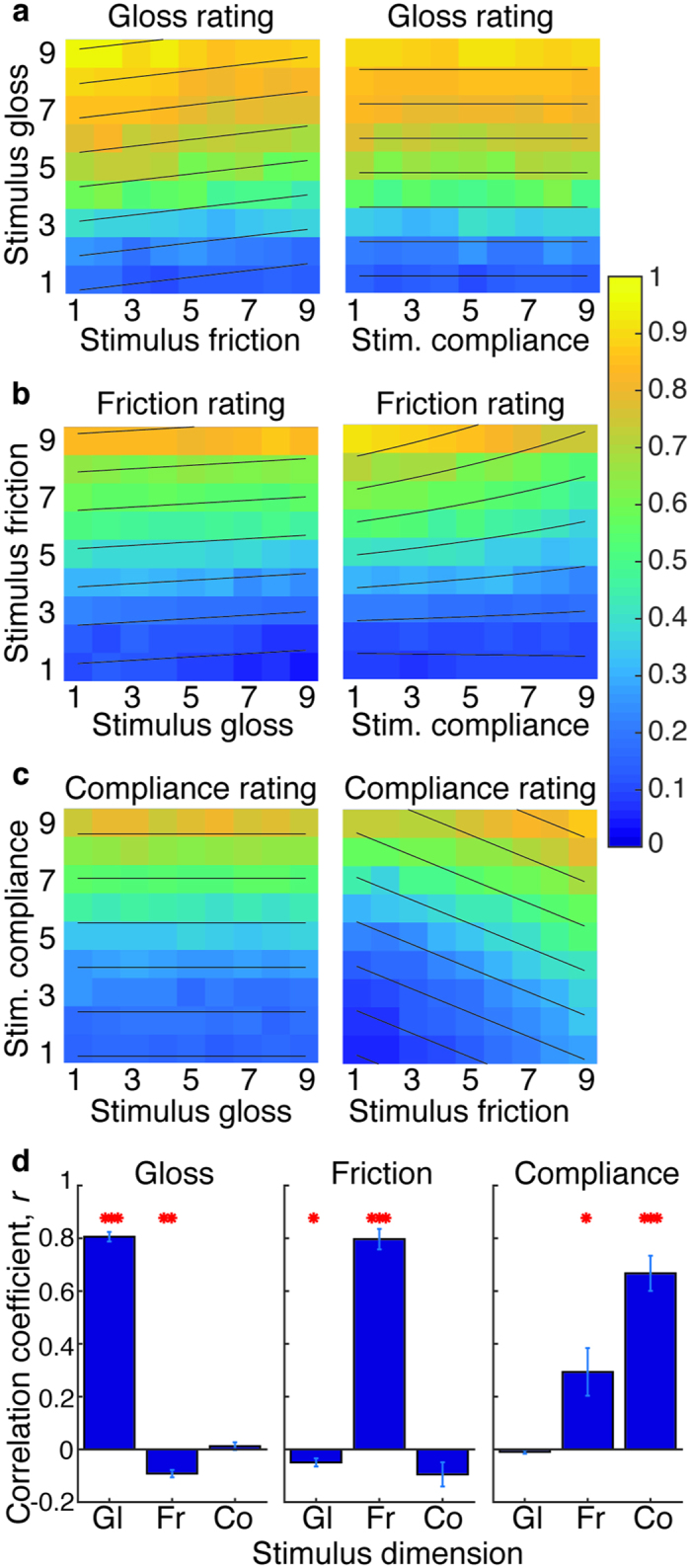
Stimulus ratings, averaged across observers, for (**a**) perceived gloss, (**b**) perceived friction and (**c**) perceived compliance. Yellow indicates high ratings; blue indicates low ratings, as indicated by the legend. Dashed contour lines show stimulus parameter pairings that produce equal ratings, as determined by the optimal regression models. (**d**) Mean correlation between each stimulus parameter (gloss: ‘Gl’, friction: ‘Fr’ and compliance ‘Co’) and each of the three rating scales (each rating type shown in a separate plot). Error bars give ± 1SE across observers. Asterisks show significant correlations, from one-sample *t*-tests against 0 (*p < 0.05, **p < 0.01, ***p < 0.001).

Figure 3a shows observers’ gloss perception as a function of stimulus gloss and stimulus friction (left plot), and stimulus gloss and stimulus compliance (right plot). The best model of perceived gloss (as determined by leave-one-out cross-validation) included both stimulus gloss and stimulus friction as predictors and accounted for an average of 66 ± 3% of response variance, across observers. As expected, perceived gloss increased significantly with stimulus gloss. However, as can be seen from Fig. 3a, the objects rated as glossiest were not only high in stimulus gloss, but also had the lowest friction, although the effect of changing friction was smaller –11% (on average, across subjects) of the effect of changing stimulus gloss.

Stimulus compliance had no significant effect on perceived gloss. Could this be because compliance is a less reliable cue than gloss or friction in this study? The correlation between stimulus compliance and compliance ratings (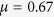) is weaker than the parallel correlations for the other two attributes (stimulus gloss vs. gloss rating: 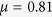, stimulus friction vs. friction rating: 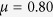, see Fig. 3d), suggesting that compliance is a less reliable signal, and would therefore have a smaller weight if gloss and compliance were (partially) integrated. However, by the same logic, gloss (a more reliable signal) would be expected to have a relatively large influence on perceived compliance, if the two were integrated. This is not evident in the data: neither the correlation between stimulus compliance and perceived gloss, nor the correlation between stimulus gloss and perceived compliance are significantly different from 0. It seems unlikely, therefore, that the lack of interaction between compliance and gloss is due to reliability differences. Rather, our data suggest, in agreement with a previous study^34^, that observers have not learnt a significant correlation between compliance and gloss from their normal interactions with the world.

The gloss ratings are in agreement with the discrimination data from Experiment 1 – observers appear to have learnt a relationship between how glossy something looks, and how it feels – glossier objects feel less ‘rubbery’, and this is driven by friction – glossier objects tend to be more slippery.

#### Friction ratings

Perceived friction was best predicted by stimulus friction, stimulus compliance, a friction x compliance interaction and stimulus gloss. This model explained an average of 67 ± 6% of the variance in friction responses, across observers. There was a small but significant effect of stimulus gloss on perceived friction: glossy objects were perceived as having lower friction: the difference in friction rating from minimum to maximum stimulus gloss was only 3.5% of the full rating scale, averaged across observers.

The intra-modal interactions between friction and compliance, although not directly relevant to our research question, are interesting nonetheless. Compliance had a small but significant effect on perceived friction: more compliant objects were perceived as more slippery (the correlation between compliance and perceived friction did not reach significance – see Fig. 3d, but compliance was a significant predictor alongside other terms in the regression model). Compliance could affect perceived friction in this way if observers were partially confusing lateral and tangential finger displacements. Further, in the physical model implemented in the Phantom, the frictional force is proportional to the force in the normal direction (see supplementary information for details of the force models).

#### Compliance ratings

As noted above, gloss and compliance appear to be processed independently; compliance ratings were independent of stimulus gloss. However, interactions between compliance and friction were also apparent within compliance ratings – these were best predicted by a model that included both stimulus compliance and stimulus friction, accounting for 59 ± 5% of response variance, averaged across observers. Objects that were physically compliant, but also had high friction were rated as most compliant. The effect of stimulus friction was large: around 40% of the effect of changing stimulus compliance. The reason for this interaction is not clear – when objects have high friction the finger doesn’t slip across the surface - introspection suggests that in this case the observer is confident that they are moving the finger only in the surface normal direction, and that this creates the impression of increased compliance (this might be also be conceptualised as a contrast effect between normal and tangential resistance). We note, however, that the positive relationship between stimulus friction and perceived compliance is hard to reconcile with the negative relationship between perceived friction and stimulus compliance – further work is required to better understand these interactions.

## Discussion

We show that observers have learnt a statistical relationship between the material cues of visually-defined gloss and haptically-defined friction and that knowledge of this statistical relationship is reflected in the integration of these cues.

Experiment 1 used a discrimination paradigm to demonstrate that observers partially integrate visual and haptic material cues: when a standard stimulus was compared to an object that was visually more glossy, but felt more rubbery, the difference was hard to detect. In contrast, an increase in visual gloss accompanied by a decrease in rubberiness was easier to detect.

Experiment 2 confirmed that friction, rather than compliance, is the haptic dimension that interacts with visual gloss. The pattern of discrimination seen in Experiment 1 was reflected in subjective ratings of gloss and friction: slippery objects are perceived to be glossier, and glossy objects are perceived to be more slippery.

Importantly, visual and haptic cues were uncorrelated across our experimental trials. Our observers did not, therefore, learn their visual-haptic associations in the laboratory. Rather, it seems that observers have learnt, from a lifetime of interactions with the environment, to associate, and thus partially integrate, visual gloss and haptic friction signals. That this association is learnt (rather than hard-wired) seems likely given previous demonstrations of relatively fast learning about the statistics of the environment^32^,^35^,^36^,^37^.

Is there an ecological basis for this integration? Our rating data suggest that the effect of haptic friction on perceived gloss is larger than vice versa (correlation between stimulus friction and perceived gloss: 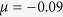, compared to correlation between stimulus gloss and perceived friction: 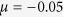), although this difference did not reach significance. The visual image is inherently ambiguous, with visual gloss cues affected not just by the specular components of the surface reflectance function, but also by object shape, motion, and the illumination environment^7^,^38^,^39^,^40^,^41^,^42^. An optimal perceptual system should, therefore, exploit all available information. However, the physical relationships between gloss and friction are complex: Decreasing surface roughness at the micro level (e.g. by polishing) can both increase gloss and decrease friction. At this scale, smoothness modulates friction by altering the contact area between surfaces^43^. However, friction and gloss can be unrelated in organic structures, such as the nanostructure that controls the low reflectance of moth eyes^44^ or the glossiness of feathers^45^. Moreover, the predominant determinant of friction for many solid surfaces may not be roughness, but adhesive forces between thin adsorbed films on solid surfaces^43^.

One of the main behavioural advantages of learning the relationship between friction and gloss may be identification of lubricant surface coatings. Whilst lubricants can be powdery and matte, they are more often water or oil-based, and highly glossy. Observers appear to use gloss in assessing the slipperiness of a surface^46^,^47^ - there are obvious advantages to identifying a wet, slippery floor. Thus, although friction is not a perfect predictor of gloss across all natural objects, the perceptual system appears to have an implicit understanding of the probabilistic relationship between these variables, and uses this to inform estimates of gloss.

## Methods

### Apparatus and stimuli

Our experimental set-up (depicted in Fig. 1a) allowed concurrent and spatially aligned visual-haptic stimulus presentation, centred at 57cm from the observer’s eyes. Head position was maintained using a headrest, and observers wore an eye patch to eliminate binocular cues to shape and gloss^8^.

Stimulus objects were spherical meshes, deformed by 3D simplex noise^48^, to produce potato-like shapes. These objects were rendered for visual presentation using an unbiased, physically-based renderer (Octane Render; Otoy Inc.), under one of three complex light fields (Beach, Campus and Uffizi^49^, see Fig. 1B). Stimuli subtended an average visual angle of 8.9°. Visually defined gloss was manipulated by varying the proportion of light reflected specularly, and the degree of specular scatter (micro-scale roughness). Additional examples of the visual stimuli can be found in the supplementary information.

The same shapes were rendered haptically using the OpenHaptics toolkit (Geomagic, USA) and presented via a Phantom force feedback device. We manipulated how ‘rubbery’ an object felt by modulating compliance-related forces in the surface normal direction (i.e. how the object responds when it is poked or squashed), and in the tangential direction (how easy it is to slide your finger across the object’s surface: static and dynamic friction). More rubbery objects were more compliant (squashy) and had higher friction. Table 1 shows the stimulus parameters for the uni-modal task of Experiment 1.

### Procedure: Experiment 1

#### Uni-modal

**Figure 4:**
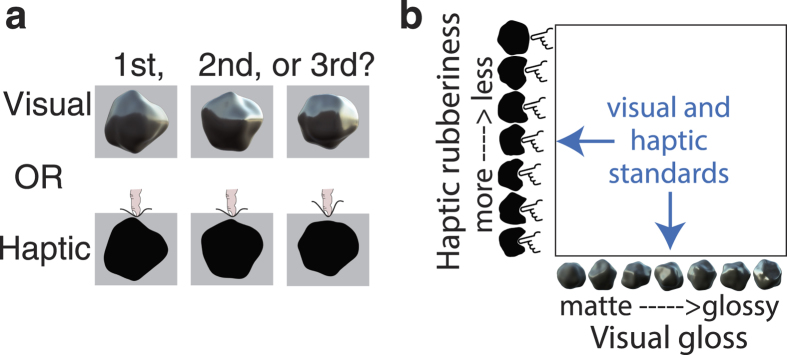
Visual and haptic uni-modal discrimination trials were used to measure each observer’s uni-modal JNDs, and thus define the visual and haptic stimulus parameter space to be used for bi-modal trials.

For each participant we first established uni-modal sets of (i) visual and (ii) haptic stimuli that were equated in terms of discriminability, via an odd-one-out task with uni-modal stimuli (Fig. 4). On each trial, three stimuli were displayed in succession (2500 msec for each stimulus): either two standard stimuli and one comparison stimulus, or one standard stimulus and two (parameter-matched) comparison stimuli. Observers reported (by using the Phantom to press a visual/haptic button rendered in the workspace) which stimulus was “made of something different”. To avoid simple image matching on visual-only trials, objects within a trial had different shapes, but all three were rendered under the same light field. In visual-only trials a virtual wall blocked haptic access to the stimuli. In haptic-only trials, a visual silhouette aided object localisation whilst eliminating visual cues to the object’s material. Observers completed 624 trials (12 comparison levels × 2 modalities × 26 repetitions) in random order, across 3 sessions of approximately 45 minutes each.

#### Bi-modal

Uni-modal discrimination data were used to create a set of 11 visual and 11 haptic stimulus levels that were (i) linearly spaced in JNDs and (ii) spanned a range of ±2 uni-modal JNDs (Just-Noticeable-Differences) around the visual and haptic standards (see Fig. 4b, fewer stimulus levels have been shown in this schematic). Gaussian distributions were fitted separately to each observer’s visual and haptic uni-modal data (fits were constrained to have a mean of 0, but standard deviation varied across observers). As part of this fitting process, stimulus index (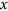) was transformed according to Equation 1, selecting exponent *p* that minimised residual error in the subsequent Gaussian fit, effectively linearising the visual and haptic parameter scales in (JND) space.


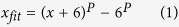


Visual and haptic stimulus parameters were then combined in two different ways, to create Type A visual-haptic stimuli and trials, in which more matte-looking stimuli felt more rubbery, or Type B stimuli and trials, in which glossier stimuli felt more rubbery, as shown in Fig. 3B. Each observer completed at least 10 blocks of 40 visual-haptic discrimination trials presented in a random order, making a 3IFC decision on each trial to identify the odd-one-out that was “made of something different”. (Due to a programming error, a subset of observers had one condition missing from initial blocks, and completed an additional 10 blocks). The same instruction was given across all uni-modal and bi-modal trials in order to (i) make it clear that 3D shape was irrelevant to the task, and (ii) to avoid biasing the observers to rely on one modality more than the other on bi-modal tasks. Each visual-haptic object was initially presented as a silhouette until the observer made haptic contact, from which point it was viewed and explored haptically for 2500 msecs. Similarly to the uni-modal case, the odd-one-out could be either a standard, or a comparison stimulus. Type A and B trials were randomly intermingled.

#### Integration and bi-modal discrimination performance.

**Figure 5:**
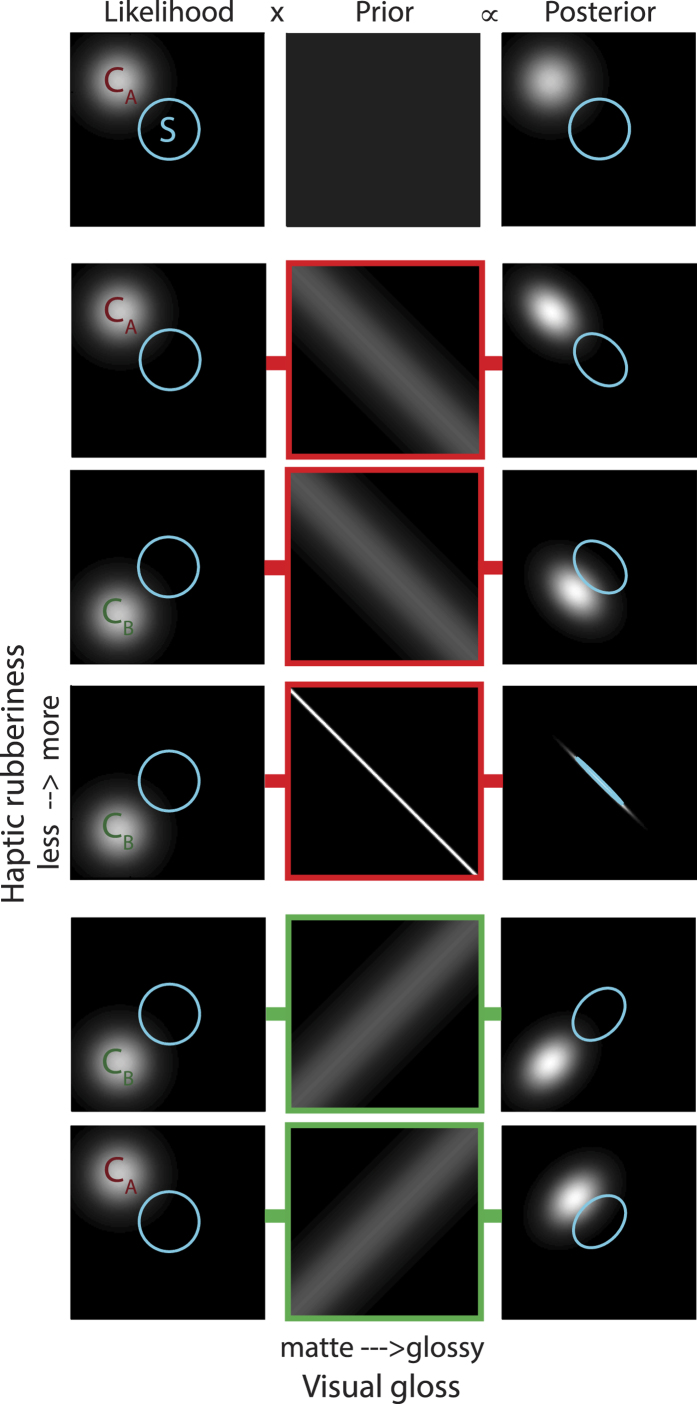
Visual-haptic integration with different coupling priors. Each row shows a likelihood (left column: the information associated with a particular visual-haptic signal), a coupling prior (middle column) and their product, the posterior (right column). Coupling priors in different rows reflect (i) no relationship between visual gloss and haptic rubberiness (top row), or (ii) a weak (rows 2–3) or strong (row 4) negative correlation or (iii) a weak positive correlation (rows 5–6). The effect of different coupling priors can be seen for stimuli lying on the axis of the negative prior (such as C_A_), or lying on the axis of the positive prior (C_B_). For comparison, the blue ring represents a ‘standard’ stimulus – an object in the middle of the visual-haptic space.

If observers have learnt that visual gloss and haptic rubberiness signals are correlated in the environment, the signals should be integrated. Integration will modify estimates of gloss and rubberinesss and discriminability of visual-haptic stimuli that vary along these dimensions. Knowledge about the statistical relationship between sensory signals can be represented by a coupling prior^32^, where the width of the prior corresponds to the strength of the correlation, and the associated degree of integration. Figure 5 shows optimal sensory integration under different hypothetical coupling priors.

The 2D Gaussian in the top left plot of Fig. 5 represents the sensory likelihood for a single object (comparison stimulus C_A_) that is visually matte and feels rubbery. If visual gloss signals are statistically unrelated to how an object feels, our perceptual system should have learnt a uniform (flat) coupling prior (Fig. 2, top row) and these visual and haptic dimensions would be processed independently, with the prior having no effect on the final visual and haptic estimates represented by the posterior (right column).

Alternatively, humans may have learnt that matte objects are more likely to feel rubbery, whereas glossy objects more often feel hard and slippery (red boxes); this possibility is shown in rows 2–4, for a weak negative correlation (rows 2–3; the spread, 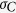, of the coupling prior shown is twice that of the uni-modal likelihoods) or a near perfect negative correlation (row 4). For a stimulus on the axis of the coupling prior (C_A_, row 2), integration does not change the mean visual and haptic estimates (likelihood and posterior distributions are aligned), but noise is reduced in the direction orthogonal to the prior. In this case, discriminating between this stimulus and the standard stimulus (S, blue circle) will be very slightly easier than if the two signals were processed independently, i.e. under a uniform coupling prior (row 1).

We can now consider a stimulus that is visually matte but does not feel rubbery – instead it feels hard and slippery like ice (Stimulus C_B_, row 3). Under the weak negative coupling prior, integration shifts the visual and haptic estimates towards the prior, and distinguishing this stimulus from the standard stimulus becomes harder. With the strongest coupling prior (row 4) the visual and haptic estimates are fully integrated; the posterior distributions for the stimuli C_B_ and S – whose likelihoods were separated in the direction orthogonal to the prior - become superimposed and discrimination between them becomes impossible – they are visual-haptic metamers. In other words, a negative coupling prior, representing a negative correlation between gloss and rubberiness, will result in better performance (smaller discrimination thresholds) for Type A trials than for Type B trials.

In contrast, we may have learnt a statistical relationship between signals such that glossy objects tend to feel more rubbery (green boxes). This situation is shown in the last two rows of Fig. 5. Given this positive coupling prior, integration results in poorer discrimination of C_A_ from S, but slightly improved discrimination of C_B_ from S, relative to independent processing of the two stimuli. In other words, a positive coupling prior will lead to larger thresholds for Type A, than Type B trials.

**Figure 6:**
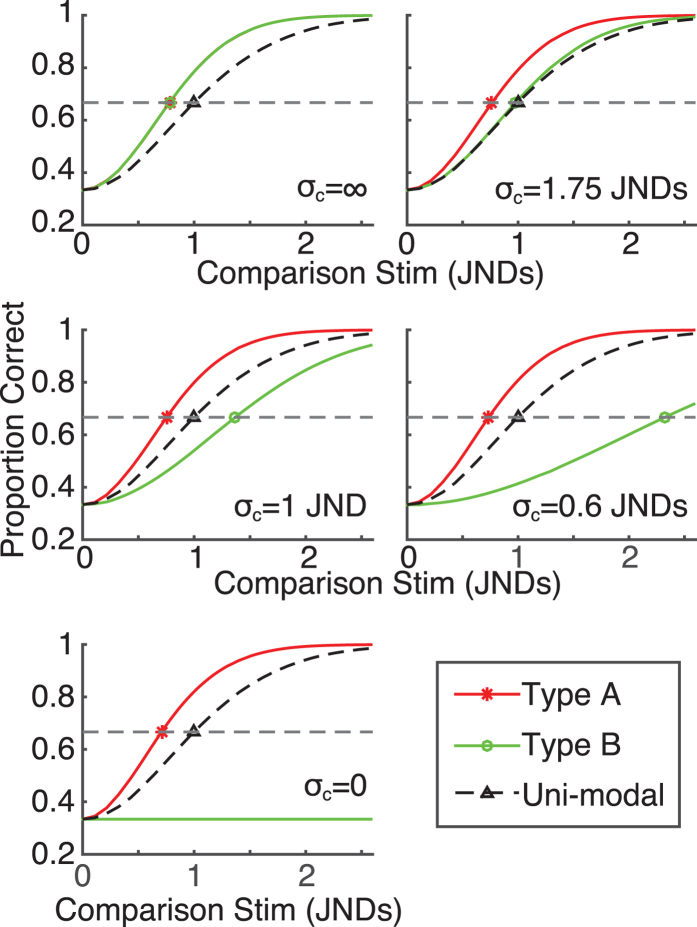
Predicted discrimination performance, given a negative coupling prior. The proportion of correct trials is shown as a function of the distance between standard and comparison stimuli in units of uni-modal JNDs. Each plot shows a different coupling prior, varying from infinitely broad (no integration) to infinitely narrow (full integration).

Figure 6 shows how discrimination performance for Type A (red) and Type B (green) stimuli is modulated by the strength of integration (i.e. the spread, 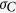, of the coupling prior). Without integration (top left plot, 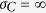), the discrimination threshold (defined by 67% correct performance – dashed grey line) is the same for both trial types, and equal to 0.78 uni-modal JNDs. Note that in our paradigm we don’t expect the usual two cue advantage of 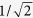 (relative to a uni-modal threshold of 1 JND). This is because our observers are performing a bivariate discrimination task: their visual-haptic estimates are subject to noise in two dimensions (as represented by the Gaussian blobs in Fig. 5). If our observers knew, on each trial, whether the stimuli differed along the positive or negative axis and could discount noise in the orthogonal (irrelevant) direction, we would predict thresholds of 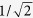 in the absence of integration.

For the negative coupling prior modelled in Fig. 6, as the spread of the coupling prior decreases, Type A thresholds decrease and Type B thresholds increase. The top right plot shows the prediction for a coupling prior whose spread is 1.75 times that of the unimodal likelihoods. In this case the Type B threshold is around 1JND, and the Type A threshold is close to 0.8 JNDs. This is comparable to our observers’ thresholds (see Fig. 2c). Under full integration (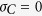, as in standard Bayesian integration models), as depicted in the bottom panel of Fig. 6, the Type A threshold is 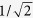 and Type B stimuli become indistinguishable.

### Procedure, Experiment 2

Observers were presented with a single object on each trial to view and touch. Its visual gloss, haptic compliance, and haptic friction (as well as its shape) varied independently from trial to trial. As in Experiment 1, the visual stimulus appeared as a silhouette until the observer made haptic contact with it. After viewing and haptically exploring the stimulus for 2500 msec., the observer rated it on the dimensions of gloss (from ‘most matte’ to ‘most glossy’), compliance (‘most hard’ to ‘most squashy’) and friction (‘most slippery’ to ‘most sticky’). Responses were given by moving virtual visual-haptic markers along three slider bars, which had linearly spaced tick marks corresponding to steps of 0.1 on a scale of 0 to 1. Before these experimental trials, and at the beginning of each session, each observer was given unlimited time to explore three pairs of reference objects to acquaint themselves with the rating scales: (i) a pair of visual objects (no haptics) that represented the highest and lowest gloss values, labelled ‘most matte’ and ‘most glossy’, (ii) a pair of visual-haptic objects that were shown in silhouette, with mid-range friction, representing the most and least compliant values, labelled ‘hardest’ and ‘most squashy’ (iii) a pair of silhouetted objects, with mid-range compliance, with the lowest or highest friction values of the stimulus set, labelled ‘most slippery’ to ‘most sticky’. The lowest and highest parameter values were the same as those used in the odd-one-out experiment, and shown in Table 1.

Six observers (2 authors, 4 naive) each completed 1458 trials (9 gloss levels x 9 compliance levels × 9 friction levels × 2 light field illuminations: ‘Beach’ and ‘Campus’ from the Debevec set^49^ split across multiple sessions of around 25–35 minutes each, with the total participation time of approximately 2 hours. Both experiments were approved by the University of Southampton Ethics Committee and were conducted in accordance with the University of Southampton’s policy on the ethical conduct of research and studies involving human participants. All participants gave informed written consent.

## Additional Information

**How to cite this article**: Adams, W. J. *et al.* Touch influences perceived gloss. *Sci. Rep.* **6**, 21866; doi: 10.1038/srep21866 (2016).

## Supplementary Material

Supplementary Information

## Figures and Tables

**Table 1:**
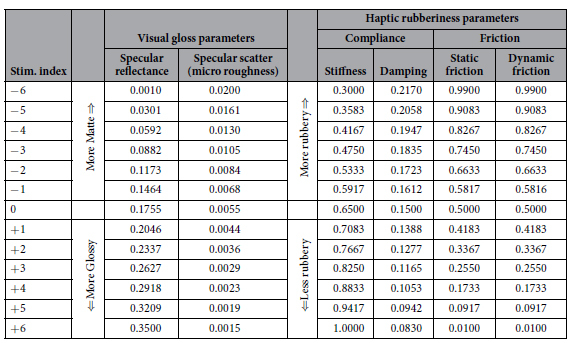
Visual and haptic stimulus parameters.

Shaded cells show the parameters of the standard stimuli used in both uni- and bi-modal trials.
