# Supplementary material for: Touch influences perceived gloss
Source: Sci Rep. 2016 Feb 26;6:21866. doi: 10.1038/srep21866 (PMC4768155; doi:10.1038/srep21866)
Supplement: Supplementary Information [file srep21866-s1.pdf]

TOUCH INFLUENCES PERCEIVED GLOSS:

SUPPLEMENTARY INFORMATION

WENDY J. ADAMS, IONA S. KERRIGAN, ERICH W. GRAF

Psychology, University of Southampton, Southampton, SO17 1BJ, ENGLAND

Corresponding author:

Wendy J. Adams
Psychology, FSHS
University of Southampton
Southampton, SO17 1BJ
ENGLAND

Tel: +011 (44) 0238059 3629
Email: wendya@soton.ac.uk

Haptic force models

Haptic forces were generated by a PHANToM Premium 1.5 HF force-feedback device (Geomagic, USA). The PHANToM device has a maximum exertable force of 8.5N, though in our experiments the maximum force was capped at 1.0N. We used the OpenHaptics software toolkit to specify the haptic scene geometry and to simulate varying haptic material properties of our stimulus objects.

As indicated in the main text and Table 1, we created haptic objects that differed in compliance and friction. These haptic dimensions were varied using the OpenHaptics Haptic Library API (HLAPI).

1. ***Compliance-related parameters***
   Different levels of compliance were created by modified two parameters: stiffness and damping (see Table 1). The stiffness parameter, k, specifies a force F_N in the surface normal direction, as defined by Hooke's Law: $F_N = kx$, where x is displacement in the normal direction. Thus, in this spring force model, k determines how aggressively a spring tries to restore itself to its rest state. For large values of k an object has low compliance and feels hard. In the HLAPAI, stiffness can take any value between 0 and 1, where 0 represents a surface with no resistance and 1 represents the stiffest surface that the haptic device can stably render.

   The damping parameter, b, adds a velocity-dependent component to forces in the normal direction, such that $F_N = kx + bv$, where v is the velocity of the end-effector. The damping parameter can take values between 0 (no damping) and 1 (the maximum damping the haptic device is capable of rendering).

2. ***Friction-related parameters***
   We modified two friction-related parameters in the HLAPI: static and dynamic friction in a 'stick-slip' friction model. The friction force, F_T, opposes motion across the object's surface, i.e. in the surface tangent direction and is directly proportional to the force in the surface normal direction. The static friction parameter determines the tangential force experienced as the device transitions from static to moving. Dynamic friction is experienced as the device moves along a surface. In our experiments static (t_s) and dynamic (t_d) friction parameters were always equal, such that $F_T = tF_N$. For either friction parameter, a value of 0 relates to a completely frictionless surface and a value of 1 corresponds to the maximum friction that the device is capable of rendering.

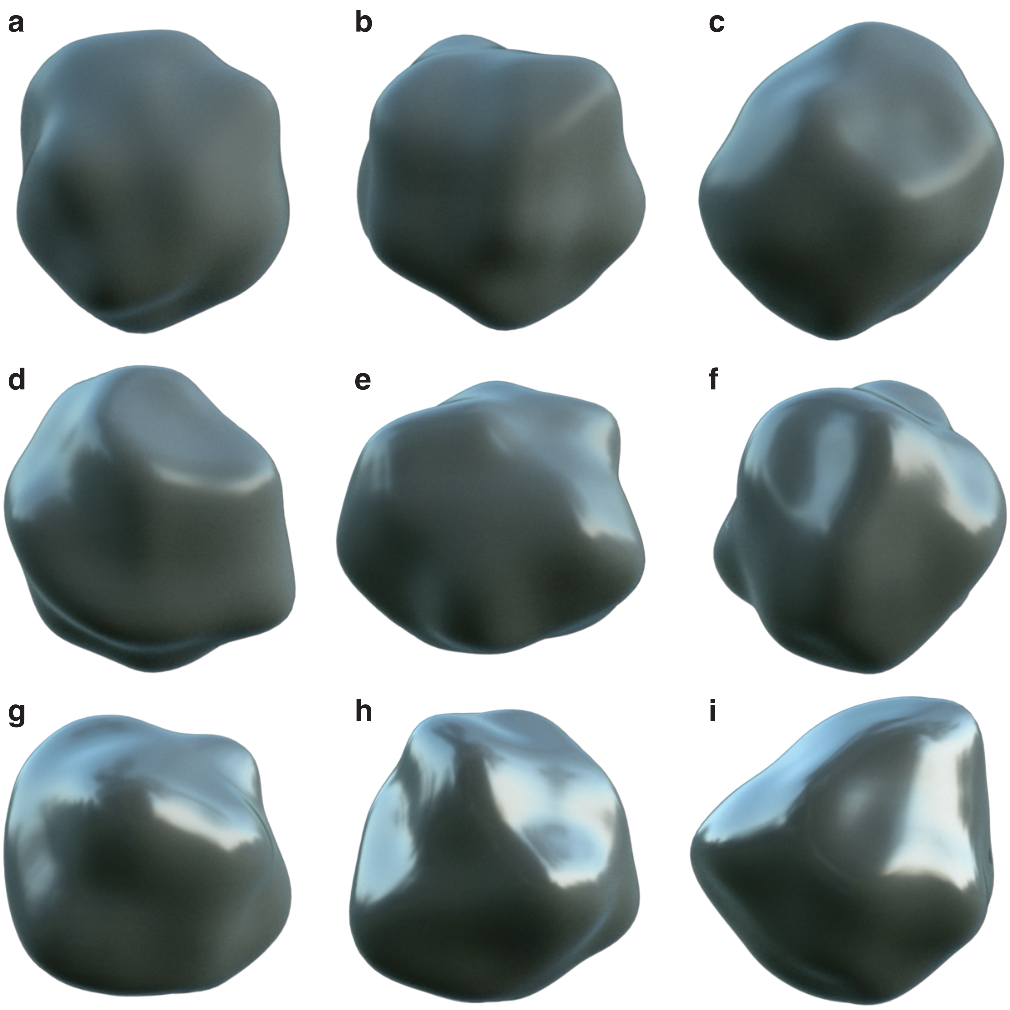

Figure S1. Example visual stimuli showing the full range of gloss values presented in our experiments. The 9 stimuli shown here correspond to the 9 gloss levels of the rating task in Experiment 2. (a) The least glossy stimulus value used in Experiments 1 and 2; stimulus index -6 in Table 1, corresponding to gloss level 1 in Experiment 2. (e) A mid-range gloss stimulus, corresponding to the visual standard in Experiment 1 (stimulus index 0 in Table 1) and gloss level 5 in Experiment 2. (i) The glossiest stimulus value in Experiments 1 and 2 (stimulus index +6 in Table 1), corresponding to gloss level 9 in Experiment 2.
